# Mapping the Surveillance Data Infrastructure in the U.S. for SARS-CoV-2, Influenza, and Respiratory Syncytial Virus

**DOI:** 10.1007/s40471-026-00405-w

**Published:** 2026-08-21

**Authors:** Akshay Deverakonda, Emily E. Ricotta, John T. Kubale

**Affiliations:** 1 Department of Epidemiology, Rollins School of Public Health, Emory University, Atlanta, GA USA; 2 Department of Preventive Medicine and Biostatistics, Uniformed Services University of the Health Sciences, Bethesda, MD USA; 3 ICPSR, Institute for Social Research, University of Michigan, Ann Arbor, MI USA

**Keywords:** Disease surveillance, Surveillance data, Influenza, RSV, SARS-CoV-2, Data infrastructure

## Abstract

**Purpose of Review:**

Infectious disease surveillance forms the epidemiological bedrock of public health, supporting the continuous and systematic collection, and analysis of health data. However, these data are collected by a fragmented network of systems and entities which complicate their accessibility and efficient use. This review describes the key surveillance systems tracking three important respiratory viruses: influenza, respiratory syncytial virus (RSV), and SARS-CoV-2; highlights these systems’ limitations; and identifies opportunities for improvement.

**Recent Findings:**

High quality surveillance data are vital for effective public health interventions. The decentralized nature of the U.S. public health infrastructure and the subsequent patchwork of surveillance networks create a formidable challenge for epidemiologists seeking to mobilize these data for outbreak response, policy evaluation, or population-based research. Greater clarity on these key surveillance systems, the data they collect, and the populations they represent could support their more effective use to support public health research and emergency response.

**Summary:**

We identified thirteen surveillance data sources monitoring influenza, RSV, and SARS-CoV-2 in the U.S; all but one were governmental. Most (9) were case-based systems, 2 were syndromic surveillance systems (that monitor for increases in illness-associated symptoms in a population rather than the disease itself), and 2 were classified as “non-traditional” sources like wastewater surveillance systems. We also assessed the relative accessibility of data from these systems and of data from state and local entities which often feed into these systems, and collated this information to improve its findability and accessibility.

**Supplementary Information:**

The online version contains supplementary material available at 10.1007/s40471-026-00405-w.

## Introduction

Disease surveillance forms the epidemiological bedrock of public health, providing the continuous, systematic collection and analysis of health data [1]. These data allow us to monitor the overall burden of disease, locate communities and populations at increased risk, and for infectious diseases, understand disease transmission dynamics and identify emerging or re-emerging pathogens [1, 2]. The existing U.S. public health system, and thus the disease surveillance infrastructure, is a mosaic of overlapping, independently operated programs spanning federal, state, and local jurisdictions [3–5]. Across this landscape, data are collected through a diverse array of mechanisms, including passive and active surveillance, sentinel networks, laboratory-based reporting, syndromic systems, and wastewater sampling [6–8]. Each of these are designed with distinct objectives, case definitions, reporting timelines, and levels of geographic resolution. While this heterogeneity reflects the decentralized nature of the U.S. public health infrastructure and the historical evolution of disease-specific reporting priorities, it presents a formidable challenge for epidemiologists seeking to mobilize these data for outbreak response, policy evaluation, or population-based research [5]. Understanding where these data live, who curates them, and what questions they are and are not designed to answer is a prerequisite for their rigorous use.

In this paper we will describe key surveillance systems used to monitor three important respiratory viruses: influenza, respiratory syncytial virus (RSV), and SARS-CoV-2. These pathogens are each substantial drivers of morbidity and mortality in the U.S. with yearly hospitalizations from each measured in the hundreds of thousands [9, 10]. Their outsized impact on population health underscores the fundamental importance of quality surveillance data. As such, we also endeavor to highlight limitations of existing systems and identify potential opportunities for improvement.

## Methods

As a first step, we conducted an environmental scan [11, 12] of surveillance systems housed at the U.S. Centers for Disease Control and Prevention (CDC) for monitoring the three respiratory pathogens of interest. Focusing on systems at the federal level allowed us to characterize how locally collected data are aggregated to create national-level estimates and also captures the most frequently used sources for obtaining these data by the public. To identify these systems, we searched the CDC website for surveillance data related to influenza, RSV, and/or SARS-CoV-2.

To identify non-CDC surveillance systems, we conducted targeted web searches, repeating this strategy until no new systems were identified. The primary search was conducted by AD, and results were iteratively reviewed as a group with all co-authors. Searches included terms and phrases like, “influenza”, “RSV”, “SARS-CoV-2”, “acute respiratory infections”, and “ARI” combined with terms like “surveillance”, and “data”. Given the incredible variability of surveillance data collection and management at the state level we leveraged a large language model (Claude, Anthropic, Opus 4.7) to conduct an initial scan of surveillance data available at state health departments. This list was then corrected and validated by hand. The full query can be found in the Supplemental Material. For each database identified, the following information was collected: source name, pathogen(s) captured, type of surveillance data, collection method, temporal and geographic resolution, available data formats, data access restrictions, and update frequency (Supplemental Table 1).

In addition to primary surveillance databases, we located several platforms that aggregated data from other sources (i.e., CDC, Google Trends). While these are incredibly useful tools for researchers and others to access surveillance data, we consider them data aggregators and not true surveillance systems for the purposes of this review (Supplemental Table 2). Similarly, non-governmental disease surveillance systems focused on only a single geographic area (e.g., GermWatch [13]) were also excluded from this review, focusing instead on the most impactful systems and those collected by state public health agencies—which already display a great deal of heterogeneity.

## Results

We identified a total of thirteen surveillance data sources (Tables 1 and 2) that monitor influenza, RSV, and SARS-CoV-2 in the U.S. All but one (WastewaterSCAN) [14] were governmental, involving CDC and other governmental public health entities [15–25]. Ten sources captured SARS-CoV-2, 10 captured influenza, and seven captured RSV (Table 1). Generally, all sources featured both current and historical surveillance data. Most surveillance sources featured both a dashboard and downloadable data, and the geographic scale of each source ranged from individual surveillance sites to national-level aggregate reporting. Nine sources had publicly available data (Table 2).

**Table 1:** Overview of key surveillance systems for influenza, RSV, and SARS-CoV-2

Source	Influenza	RSV	SARS-CoV-2	Case-based	Syndromic	Non-traditional	Active vs. Passive	Data collected
National Syndromic Surveillance Program	Yes	Yes	Yes		x		Passive	ED^a^ visits, viral activity, test positivity
CDC Wastewater Monitoring Program	Yes	Yes	Yes			x	Passive	WW^b^ surveillance
WastewaterSCAN	Yes	Yes	Yes			x	Passive	WW^b^ surveillance,
ILINet	Yes	No	No		x		Passive	ILI^c^ visits
NCHS Pneumonia & Influenza Mortality	Yes	No	Yes	x			Passive	Influenza-confirmed mortality
NVSS Provisional COVID-19 mortality surveillance	No	No	Yes	x			Passive	Provisional death counts
New Vaccine Surveillance Network (NVSN)	Yes	Yes	Yes	x			Active	Viral trends at sentinel sites
National Respiratory and Enteric Virus Surveillance System (NREVSS)	Yes	Yes	Yes	x			Passive	LC^d^ tests
FluSurv-NET	Yes	No	No	x			Active	LC^d^ influenza-associated hospitalizations
RSVNet	No	Yes	No	x			Active	LC^d^ RSV hospitalizations
COVID-19 Net	No	No	Yes	x			Active	LC^d^ COVID-19 hospitalizations
National Healthcare Safety Network	Yes	Yes	Yes	x			Passive	Weekly hospital admissions
National Notifiable Diseases Surveillance System	Yes	No	Yes	x			Passive	Influenza-associated pediatric mortality, novel influenza A infections, covid-19 infection, covid-19 associated pediatric mortality

^a^Emergency department

^b^Wastewater

^c^Influenza-like illness

^d^Laboratory-confirmed

**Table 2:** Features data from key surveillance systems

Source	Timeframe	Format	Geography	Publicly accessible?	Lag
National Syndromic Surveillance Program	N/A^a^	Downloadable	National, state (contributing hospitals)	Yes^a^	Updated weekly^a^
CDC Wastewater Monitoring Program	COVID (January 2020), Influenza A (September 2021), RSV (February 2022)	Dashboard & Downloadable	National & state level	Yes	Updated weekly
WastewaterSCAN	January 2023 - present	Dashboard	Individual wastewater treatment plants nationwide	No	Updated weekly
ILINet	2008 - present	Dashboard & Downloadable	State & Core-Based Statistical Areas (CBSA, up to 945)	Yes	Updated weekly
NCHS Pneumonia & Influenza Mortality	2017-present	Dashboard & Downloadable	National, HHS Region, State	Yes	Updated weekly
NVSS Provisional COVID-19 mortality surveillance	2020-present	Dashboard & Downloadable	National and State	Yes	Updated weekly
New Vaccine Surveillance Network	2017 - present	Dashboard & Downloadable	7 sentinel surveillance sites (Vanderbilt University Medical Center, Nashville, TN, University of Rochester School of Medicine and Dentistry, Rochester, NY, Cincinnati Children’s Hospital Medical Center, Cincinnati, OH, Texas Children’s Hospital, Houston, TX, Seattle Children’s Hospital, Seattle, WA, Children’s Mercy Hospital, Kansas City, MO, Children’s Hospital of Pittsburgh, Pittsburgh, PA)	Yes	One month
National Respiratory and Enteric Virus Surveillance System (NREVSS)	2019-present	Dashboard	National, HHS region	No	Updated weekly
FluSurv-NET	2003 - present	Dashboard & Downloadable	Specific counties in Georgia, North Carolina, Tennessee, Maryland, New York, Connecticut, Michigan, Minnessota, Utah, Colorado, New Mexico, California, Oregon, Washington	Yes	Three weeks
RSVNet	2016 - present	Dashboard & Downloadable	Specific counties in Georgia, North Carolina, Tennessee, Maryland, New York, Connecticut, Michigan, Minnessota, Utah, Colorado, New Mexico, California, Oregon, Washington	Yes	Three weeks
COVID-19 Net	May 2020 - present	Dashboard & Downloadable	Specific counties in Georgia, NC, Tennessee, Maryland, New York, Connecticut, Michigan, Minnessota, Utah, Colorado, New Mexico, California, Oregon, Washington	Yes	Three weeks
National Healthcare Safety Network	2020 - present	Dashboard & Downloadable	Health care facilities nationwide	Yes	Two weeks
National Notifiable Diseases Surveillance System	2004 - present (influenza-associated pediatric mortality), 2007-present (novel influenza A infections), 2020-2025 (covid-19 infection), 2026 - present (covid-19 associated pediatric mortality)	Downloadable	Nationwide	Yes	Varies by disease

^a^Limited data (e.g., ED visits for influenza, RSV, and SARS-CoV-2) available via data.gov. Most data aren't publicly available

Of note, national-level data are most often actually collected by state, tribal, local, and territorial health entities (referred to collectively as “local” for the duration of this paper). These data are then aggregated by the CDC for disease monitoring. Some local health agencies also directly provide data or reports, but many do not, and the amount of data provided, its format, completeness, and level of documentation differ (Supplemental Table 1).

### Case-Based Surveillance Systems

Nearly 70% of the surveillance systems that we encountered collected laboratory-confirmed cases for specific pathogens (Table 1). However, there exists a great deal of within-group data variability.

One of the most important U.S. infectious disease surveillance systems is the National Notifiable Diseases Surveillance System (NNDSS) [26, 27]. This system is a joint effort between the CDC, the Council of State and Territorial Epidemiologists (CSTE), and local health departments, collecting data on approximately 120 infectious diseases indicators. The most robust data exist for conditions classified as federally “notifiable”, meaning that public health agencies are strongly encouraged to notify CDC of all cases meeting the specified criteria. This differs from “reportable” conditions which are set at the state/territory level [28]. The designation of a condition as reportable carries legal weight: healthcare providers are required by law to report diagnoses to government health officials within a specified timeframe, with non-compliance subject to regulatory or financial penalty [29–31]. This enforcement structure makes notifiable disease reporting among the more consistent and dependable mechanisms in the U.S. surveillance ecosystem. However, NNDSS is not without limitations. While many “notifiable” conditions are also classified as “reportable”, this is not universal [28]. CDC and CSTE annually update the list of notifiable conditions, including specific case definitions, but participation by localities remains voluntary [32]. Additionally, the NNDSS provides a relatively narrow picture of the captured conditions. For example, it focuses more heavily on emerging pathogens with pandemic potential (e.g., novel influenza viruses), or those impacting high-risk populations (e.g., pediatric mortality) [33].

In contrast to the breadth of conditions tracked via NNDSS, other case-based surveillance systems focus on single pathogens. Given its substantial yearly burden and pandemic potential, it is perhaps unsurprising that influenza is the primary focus of one such system: FluSurv-NET [15]. FluSurv-NET collects near real-time data from “more than 90 counties and county equivalents in fourteen states that participate in the [CDC’s] Emerging Infections Program (EIP) [15, 34].” These data provide estimated weekly rates of laboratory-confirmed influenza hospitalizations. Similar systems exist for RSV (RSV-NET) [25] and SARS-CoV-2 (COVID-NET) [23], which together with FluSurv-NET, comprise CDC’s RESP-NET surveillance system [18].

Other case-based systems with a narrower scope include the National Respiratory and Enteric Virus Surveillance System (NREVSS) and the New Vaccine Surveillance Network (NVSN) [16, 35]. Since its creation in the 1980s, NREVSS has provided weekly data from participating hospitals, health departments, laboratories, and commercial entities on respiratory viruses like influenza, RSV, and SARS-CoV-2, among others. Indicators include the number of tests performed, test type (e.g., PCR or nucleic acid amplification tests [NAAT]), and the number of positive tests (Table 1). Conversely, the NVSN is a “prospective, active, population-based surveillance platform that enrolls children with acute respiratory illnesses (ARIs) at seven pediatric medical centers” in large metropolitan centers [35–37]. It collects laboratory-confirmed cases of influenza, RSV, SARS-CoV-2, and other respiratory viruses, clinical data like comorbidities, and vaccination status which is verified against state immunization records [17]. These data provide estimates of disease specific incidence rates, vaccine effectiveness, and even the identification of potential risk factors for severe disease.

A final sub-group of case-based surveillance systems include those designed to track outcomes or impacts in a specific setting. The National Center for Health Statistics (NCHS) at CDC, for example, focuses on infection-related outcomes, collecting vital statistics including deaths [21]. This includes provisional COVID-19 mortality data and mortality associated with laboratory-confirmed influenza [24, 38]. NCHS also captures pneumonia mortality, which is driven in large part by infection with influenza, RSV, or SARS-CoV-2 [39–43]. In contrast, the National Healthcare Safety Network (NHSN) focuses on infections in particular settings (i.e., acute care and long-term care facilities)(*NHSN | CDC*, 2026) [22]. Used by over 38,000 U.S. facilities in all 50 states, the NHSN provides data on hospital admissions for influenza, RSV, and SARS-CoV-2 by both age and admission level as well as bed capacity and occupancy [44, 45].

### Syndromic Surveillance

In contrast to case-based systems, syndromic surveillance systems track increases in specific illness-associated symptoms in a population [46]. Our search identified two primary syndromic surveillance systems that are particularly relevant to influenza, RSV, and SARS-CoV-2 – the CDC’s National Syndromic Surveillance Program (NSSP) and the U.S. Outpatient Influenza-like Illness Surveillance Network (ILINet) [20, 47]. The NSSP is CDC’s flagship initiative for syndromic surveillance, aggregating data from more than 7,500 health care facilities across all 50 states and from federal and local health departments [20]. A key feature of the NSSP is the online BioSense platform, a cloud-based platform that aggregates data from emergency departments, commercial laboratories, Department of Veterans Affairs’ medical data, air quality monitors, and other sources [48–51]. The Electronic Surveillance System for the Early Notification of Community based Epidemics (ESSENCE) is a secure online platform that allows BioSense users to access syndromic surveillance data for analysis [52]. The NSSP Community of Practice and Knowledge Repository provide users access to a wide range of resources and support related to NSSP and syndromic surveillance more broadly [53].

ILINet collects data from a network of outpatient healthcare providers in all 50 states, Washington D.C., Puerto Rico, and the U.S. Virgin Islands [47, 54]. Specifically, ILINet monitors for instances of influenza-like illness (ILI), defined as a fever (100 °F/ 37.8 °C or higher) plus cough and/or sore throat, with onset occurring within the previous seven days [54]. This is different from the World Health Organization (WHO) definition which requires a fever of 38° C or higher plus cough and symptom onset within ten days [55]. Despite these differences, both have been shown to be reliable predictors of influenza positivity, though their differences can limit comparisons of ILI rates in the U.S. with those of other countries [56].

An advantage of syndromic surveillance is that it can often identify local disease transmission before clinical confirmation of cases occurs [46]. However, this increase in sensitivity corresponds to a decrease in specificity. Rather than identifying just cases of influenza, for example, syndromic surveillance identifies respiratory illnesses more broadly which may be caused by influenza or any number of other pathogens.

### Non-Traditional Sources

Not all surveillance systems fit neatly into the above categories. In recent decades new approaches and novel data streams have provided practitioners and researchers with new mechanisms to track disease burden and spread. During the height of the COVID-19 pandemic, for example, wastewater surveillance, which samples municipal wastewater for viral material shed from people in the community, became a vital tool for the medical and public health community to anticipate surges in cases and hospitalizations [57–59]. The success of wastewater sampling is due in large part to the passive nature of data collection which does not require individuals to seek out (or be sought out) for testing. It also captures presymptomatic, asymptomatic, and mildly symptomatic individuals who are still shedding potentially infectious virus, providing a better overall picture of the level of disease active in a community. These advantages led to the establishment of the CDC’s Wastewater Monitoring Program (formerly the National Wastewater Surveillance System), a program that monitors wastewater for multiple pathogens, including influenza-A, RSV, and SARS-CoV-2, from almost 1300 sites across all 50 states [19]. Unique among surveillance systems we identified, wastewater surveillance is also conducted by non-governmental sources like WastewaterSCAN, a non-profit initiative that tracks 16 pathogens (including influenza, RSV, and SARS-CoV-2) at > 150 monitoring sites across the U.S [14, 60]. Like all systems, wastewater surveillance has limitations. Samples are typically collected from municipal wastewater systems, meaning that populations without this infrastructure (i.e., rural communities utilizing septic systems) are poorly represented [61, 62]. There is also variability in the type material tested (e.g., solids vs. influent) [63], normalization methods used to smooth signals [64], and varying levels of detection across pathogens [65, 66]. However, despite these limitations, wastewater surveillance represents an invaluable addition to the surveillance landscape.

Other non-traditional data have been correlated with infectious disease burden, but do not quite rise to the level of being considered surveillance systems. For example, Google Trends, which provides daily state- and national-level internet search data, has been used to track RSV, SARS-CoV-2, and long COVID, among many others, in the U.S. and globally [67–69]. Similarly, in the first years of the COVID-19 pandemic, some researchers monitored Yankee Candle reviews complaining about unscented candles, with such reviews increasing during peaks of COVID-19, likely due to the anosmia associated with early SARS-CoV-2 variants [70]. While neither of these data sources are sufficient to serve as standalone surveillance systems, they can prove useful in supplementing other data to provide additional insights into the burden and spread of infectious pathogens.

## Discussion

For this study, we conducted an environmental scan of the primary surveillance systems used to monitor influenza, RSV, and SARS-CoV-2 in the U.S. In total, we identified thirteen systems, most of which (9) were case-based surveillance; two syndromic and two non-traditional surveillance systems were also identified. These systems prospectively collect a wide range of data from diverse settings, using differing methodologies, and with varying geographic representation, all for the purposes of understanding infectious disease dynamics in the U.S.

The fragmented, complex, and interconnected nature of the U.S. surveillance data system is an inevitability that stems both from the public health system’s structure and from the multitude of functions it is expected to perform. While CDC is in many ways a national “health department”, in reality it functions quite differently than local health departments. For one, CDC generally cannot send staff to support public health at these levels without a formal invitation [71], meaning that most surveillance data are actually collected by local public health agencies or staff at the medical and laboratory facilities at which cases present. CDC’s primary role is to provide financial and technical support to these entities. In fact, nearly 80% of CDC’s appropriated funds go directly to local health departments [72]. This means that while CDC does have influence on what and how data are collected, the system itself is operationalized through sub-federal partnerships across the nation. Unsurprisingly, the structure of local health departments also varies. The Association of State and Territorial Health Officials classifies health departments into seven groups: centralized, largely centralized, shared, largely shared, mixed, largely decentralized, and decentralized [73]. Additionally, while communicable diseases remain a key focus of public health entities at all levels of government, the scope of their responsibilities has grown considerably, now encompassing chronic diseases, environmental health, injury prevention, and more. Health departments are also often tasked with inspecting and certifying a dizzying range of industries, from restaurants to health spas to tattoo parlors [74, 75]. This expanding mandate, combined with the “boom and bust” cycles characterizing public health funding, makes long-term planning, capacity building, and sustained improvements to communicable disease surveillance genuinely difficult to achieve [76]. These structural challenges are compounded by the diversity of data types being collected, methods of surveillance, and the resources required for conducting surveillance activities. The result is a vast amount of variability in geographic coverage, granularity, and reporting completeness of surveillance systems monitoring influenza, RSV, and SARS-CoV-2 (Table 2).

For example, passive surveillance data collected by hospitals and healthcare facilities for the NHSN Patient Safety Component is driven by a combination of voluntary participation, state-mandated reporting laws, and Centers for Medicare & Medicaid Services (CMS) quality reporting program incentives under the Hospital Inpatient Quality Reporting (IQR) Program [77, 78]. Facilities participate for one of three reasons: voluntarily, under state law, or to comply with CMS reporting requirements [79–81]. Continuous facility-based surveillance requires dedicated staff time, data collection infrastructure, and funding, which are not available across all institutions. Indeed, community hospitals and smaller rural facilities consistently emerge in the literature as more resource-constrained relative to larger academic medical centers, particularly regarding infection prevention staffing and informatics capacity. While the federal government supports surveillance through CDC-managed infrastructure (e.g., NHSN) and CMS incentive programs, direct operational costs are often borne by the reporting facility or state directly, which results in limited resources and competing priorities [82]. Despite these limitations, passive surveillance systems like those leveraging vital statistics, medical records, or viral concentration in wastewater, are arguably the most critical and widely-used mechanisms to track long-term trends across large geographic areas. They provide information on who is getting infected with particular pathogens, when infection rates tend to be highest, and whether illnesses are more/less severe than what has been historically documented.

Active surveillance systems, meanwhile, aim to capture every case (or other outcome), resulting in rich, detailed, complete data sources that allow researchers to explore targeted questions in a deeper way than can be managed using most passive surveillance systems. While there are many benefits to active systems, they require even more resources and generally have a more restricted scope than passive systems. For example, the NVSN collects focused, granular data on pediatric infections and vaccine effectiveness from only seven sentinel surveillance sites [36]. To capture all cases, the NVSN sites collect data via parent interviews, medical chart reviews, and universal specimen testing [17, 37]. While this results in near-complete case ascertainment, the tradeoff is that the NVSN is not well positioned to monitor long-term trends of disease across the U.S. Instead, active surveillance systems are better suited to providing important insights into questions like vaccine effectiveness and risk factors for severe disease.

Yet the scope of a surveillance system is not only shaped by its objectives or the number of sites that participate – it is also constrained by the underlying infrastructure of the data source itself. Wastewater surveillance illustrates this well. To monitor the concentration of pathogens in wastewater, samples have to be collected from a physical location for testing. These collection points and the design of municipal wastewater systems therefore directly impact what base population is represented by these samples. That these systems do not always perfectly align with policy-relevant boundaries like counties can complicate their use in shaping interventions.

While this complexity makes it challenging to understand the system as a whole, there have been positive changes to some aspects of data collection that improve its utility. Since 2016, collecting data following the “FAIR data” principles (findability, accessibility, interoperability, and reusability) has gained increasing traction as a means to improve data quality at a universal level [83–86]. There has also been an increase in efforts to co-localize data from different surveillance systems for increased findability. For example, CDC’s FluView Interactive combines data on ILI from ILINet, influenza-associated hospitalizations from FluSurv-NET, and influenza-associated mortality from the NCHS Mortality Surveillance System into a single interactive web application [87]. Similarly, non-governmental efforts like POPHIVE [88] and the Delphi EpiPortal [89] are also helping to make these data more findable, accessible, and reusable. However, additional work is needed. Findability is often impacted by the impermanent nature of state and federal websites housing surveillance data. This can be ameliorated by the adoption of permanent digital object identifiers and/or consolidation of raw data into a comprehensive platform like those listed above. In some instances, third-party volunteer networks such as the Data Rescue Project attempt to ensure public data are kept accessible long-term in public, non-governmental repositories like ICPSR’s DataLumos [90, 91]. Improvements to data interoperability – the ability to combine data from disparate sources – are particularly valuable. Through the NNDSS, for example, CDC and CSTE have harmonized a subset of around 120 notifiable measures (updated yearly) to provide a national-level perspective. Unfortunately, state participation is voluntary which limits the completeness of this endeavor. Additionally, each state/territory establishes its own list of officially reportable diseases and conditions. While there are commonalities across these lists, there is also a great deal of variability in the conditions included and how they are defined.

Another shortcoming of the current system is the inconsistency in population representativeness across all surveillance systems identified. While RESP-NET (and its component systems FluSurv-NET, COVID-NET, and RSV-NET) states that it represents > 40 million people in the U.S. (~ 12% of the current population), when explored further, it becomes clear that the data come from fewer than 200 counties in 14 states (~ 6% of counties). The proportion of each state’s population that is covered also varies widely ranging from only 9% in California and North Carolina to 71% in Minnesota. This limits the ability to make inferences about underrepresented people and communities. Voluntary participation of entities in a surveillance system presents another source of potential bias, as those who choose to provide data may be systematically different from those who do not. Finally, there are temporal reporting differences both across and within surveillance systems. There is up to a three-week delay in the reporting of hospitalizations through RESP-NET, so CDC uses a modeling tool to estimate the most recent three weeks’ hospitalization rates. Similarly, the NVSS sentinel surveillance system has up to a month lag in reporting data. This challenges the usefulness of surveillance data for informing policy development, particularly during public health emergencies like epidemics when timely interventions are most impactful in mitigating disease spread.

While we have discussed many limitations of respiratory disease surveillance data in the U.S., it is important to emphasize that the system still provides extraordinary public health value. While flawed, surveillance data are used to allocate resources, design interventions, and support decision making to improve public health every day. Indeed, work is being done to address some of the gaps in coverage and process limitations. When the NHSN was established in 2005, for example, it consisted of 300 hospitals. In the decades since, it has expanded to include more than 30,000 healthcare facilities, including “nearly every hospital, ambulatory surgery center, dialysis facility, and nursing home in all 50 states, Washington, D.C., and most U.S. territories [92].” Similarly, innovative methods of disease ascertainment have been quickly adopted and integrated into existing systems with great effect. Multiplex PCR panels have substantially increased the number of pathogens being monitored, and advances and availability of genetic sequencing technologies have improved our understanding of pathogen transmission and evolution. Wastewater surveillance has also become an increasingly important surveillance tool for infectious pathogens of all types where previously it was largely limited to enteric pathogens like polio. Disease surveillance in the U.S., like any other sector, has benefitted from both incremental advances and periods of rapid innovation, with the end result being that public health entities have more granular data on key infectious diseases than ever before. While gaps and limitations persist, the successes achieved despite the imperfect nature of disease surveillance systems in the U.S. should not be forgotten.

We sought here to locate and characterize major state, federal, and non-governmental surveillance data systems used to monitor influenza, RSV, and SARS-CoV-2 by describing their overall features including what data are collected, how they are collected, where they’re collected, and by whom. The intention of this paper is to help reduce confusion about these vital data so that researchers, practitioners, and the public may better understand their strengths and limitations. This paper is not intended to provide a comprehensive picture of all surveillance systems for these pathogens in the U.S. Other data sources like electronic medical records, for example, are discussed only in passing here. These limitations aside, the data and systems described here remain some of the most important for ongoing disease monitoring and mounting rapid public health response in times of crisis. While the complex, interconnected nature of disease surveillance can make it difficult to understand, it provides invaluable data on how a wide range of conditions are impacting communities around the country. Still, further improvements are needed and it is our hope that by better characterizing the complex web of surveillance systems, gaps and opportunities for improvement will be more easily identified.

## Key References

- Lipsitch M, Bassett MT, Brownstein JS, Elliott P, Eyre D, Grabowski MK, et al. Infectious disease surveillance needs for the United States: lessons from Covid-19. Front Public Health [Internet]. Frontiers; 2024 [cited 2026 May 29];12. 10.3389/fpubh.2024.1408193.
  - ○ This paper arose from discussions held at a 2022 Symposium “Quantitative Tools and Data Opportunities for Pandemic Surveillance and Response”. It explores lessons learned during the COVID-19 pandemic in the US and examines what decisions should be informed by surveillance data and the types of data needed to support these decisions.
- Neves A, Cuesta I, Hjerde E, Klemetsen T, Salgado D, van Helden J, et al. FAIR + E pathogen data for surveillance and research: lessons from COVID-19. Front Public Health. 2023;11:1289945. 10.3389/fpubh.2023.1289945.
  - ○ This article endeavors to apply lessons learned from the COVID-19 pandemic to propose a framework for improved pathogen surveillance to support both research and emergency response. The authors specifically highlight the importance of incorporating the FAIR + E data principles (*F*indable *A*ccessible *I*nteroperable *R*eusable and in an *E*quitable ecosystem) into such a framework.
- Hochheiser H, Kumar P. Using Surveillance Data to Estimate Infectious Disease Burden: Opportunities and Challenges. Am J Public Health. American Public Health Association; 2025;115:454–6. 10.2105/AJPH.2025.308023.
  - ○ This editorial highlights the challenges and complexities of infectious disease surveillance in the US. Specifically, it does so through the lens of a study that used a Bayesian hierarchical model to try and more accurately estimate the burden of influenza hospitalization in the US during the 2022–2023 influenza season. After highlighting the challenges faced by the authors in conducting this analysis, Hochheiser and Kumar discuss changes which may help mitigate these challenges moving forward.

## Supplementary Information

Below is the link to the electronic supplementary material.

Supplementary Material 1

Supplementary Material 2

## Data Availability

No datasets were generated or analyzed during the current study. An html version of Supplemental Table 1: Overview of State Surveillance Data on Influenza, RSV, and SARS-CoV-2 can be found at https://jkubale.github.io/state_table.html. The authors will endeavor to periodically update this table for use as a resource. Update requests and reports of broken links can be made by creating an issue via GitHub, or by reaching out to the corresponding author (jkubale@umich.edu).
