# Supplementary material for: Mapping the Surveillance Data Infrastructure in the U.S. for SARS-CoV-2, Influenza, and Respiratory Syncytial Virus
Source: Curr Epidemiol Rep. 2026 Aug 21;13(1):24. doi: 10.1007/s40471-026-00405-w (PMC13493409; doi:10.1007/s40471-026-00405-w)
Supplement: Supplementary file 1 [file 40471_2026_405_MOESM1_ESM.docx]

**Supplemental methods:**

**Claude (Anthropic, Opus 4.7, May 7, 2026) query for identifying state health department surveillance systems.**

**Query 1:**

I need a deep dive on which US states and territories have surveillance data for SARS-CoV-2, influenza, and RSV. For each database reviewed, I want to know: the source’s name, which pathogen(s) were captured, and the type of surveillance data featured (i.e., syndromic, laboratory tests, wastewater). I also want to know for each source whether it makes available current data or historical data, available data formats, geographic scale, data access restrictions, update frequency, and whether there is inclusion of external data sources.

**Output 1:**

Synthesized surveillance database architectures across eight pathogen-tracking systems

Synthesized surveillance database architectures across eight pathogen-tracking systems

Here is a deep dive across nine major U.S. surveillance databases covering SARS-CoV-2, influenza, and RSV. Below is a prose summary of the key findings organized by the dimensions you requested.

The Nine Databases

1. NHSN Hospital Respiratory Data (HRD)

COVID-19, influenza, and RSV reporting for hospitals is included in the Hospital Respiratory Data module in NHSN, operated by CDC with reporting mandated by CMS. This is the broadest hospital surveillance platform. Beginning November 1, 2024, CMS requires all acute care hospitals to electronically report information about COVID-19, influenza, and RSV, including hospitalization counts stratified by age group, ICU and inpatient bed capacity and occupancy. Data formats include CSV upload, API, and a web dashboard. The underlying dataset can be accessed and downloaded from the NHSN HRD interactive dashboards. Coverage is national across all states and territories for all CMS-certified facilities. The dashboard is updated Fridays with prior-week data. [CDC + 2](https://www.cdc.gov/nhsn/covid19/index.html)

2. RESP-NET (COVID-NET / FluSurv-NET / RSV-NET)

RESP-NET conducts surveillance through a network of acute care hospitals in select counties in 14 states and up to 194 counties for influenza, COVID-19, and RSV surveillance, covering more than 40.9 million people — approximately 12% of the U.S. population. This is population-based laboratory-confirmed hospitalization surveillance, yielding weekly rates per 100,000. Data are publicly available via an interactive dashboard and downloadable from data.cdc.gov. Historical data go back to 2003–04 for influenza (FluSurv-NET), 2020 for COVID-NET, and 2018 for RSV-NET. The 3 most recent weeks of RESP-NET data represent "nowcasts" — model-based estimates of hospitalization rates — subject to revision. [CDC](https://www.cdc.gov/resp-net/dashboard/index.html)

[CDC](https://www.cdc.gov/respiratory-viruses/data/illness-severity.html)

3. ILINet / FluView

ILINet consists of outpatient healthcare providers in all 50 states, Puerto Rico, the District of Columbia, and the U.S. Virgin Islands; more than 124 million patient visits were reported during the 2024–25 season. This is syndromic outpatient surveillance — it captures the ILI syndrome (fever plus cough or sore throat) rather than lab-confirmed pathogens. ILINet monitors outpatient visits for respiratory illness referred to as influenza-like illness, not laboratory-confirmed influenza, and will capture respiratory illness visits due to infection with any pathogen that can present with similar symptoms, including SARS-CoV-2 and RSV. Data are publicly downloadable in CSV format via FluView Interactive, updated weekly, with historical data back to 1997–98. [CDC](https://www.cdc.gov/fluview/overview/index.html)

[CDC](https://www.cdc.gov/fluview/surveillance/2025-week-52.html)

4. NSSP / ESSENCE (Emergency Department Syndromic)

NSSP is a collaboration among CDC, local and state health departments, with private sector partners; the national data include emergency departments in all 50 states, the District of Columbia, and Guam. NSSP captures diagnosed COVID-19, influenza, and RSV from ICD-10 codes submitted within 24 hours of an ED visit. More than 10.2 million electronic health messages are received by NSSP every day, with 85% of U.S. emergency departments participating. Full access requires credentialing via the ESSENCE BioSense Platform (restricted to public health professionals), but a public dashboard is freely available. Current data sharing agreements limit federal access to state and local NSSP data to only multi-state regional aggregations for most analytic purposes, which was a significant challenge during COVID-19. Jurisdiction-level public data are suppressed for Missouri and historically for several other states. [CDC + 2](https://www.cdc.gov/nssp/php/onboarding-resources/companion-guide-ed-data-respiratory-illness.html)

5. NREVSS (Laboratory Surveillance)

NREVSS was created in the 1980s; participating laboratories from university and community hospitals, state and county public health departments, and commercial entities voluntarily report weekly aggregate test counts and positive results. This is the primary lab-based system for SARS-CoV-2, influenza A/B, and RSV test positivity. Data are viewable at national and HHS region levels (not state level on the public dashboard). Results are limited to PCR/NAAT tests. CSV downloads are freely available via data.cdc.gov and healthdata.gov, updated weekly. NREVSS plays an important role in describing the temporal and geographic circulation patterns of respiratory and enteric viruses, including monitoring changes in the typical annual circulation patterns and identifying viral outbreaks. [CDC](https://www.cdc.gov/nrevss/php/dashboard/index.html)

[CDC](https://www.cdc.gov/nrevss/php/dashboard/index.html)

6. NWSS (National Wastewater Surveillance System)

CDC's Wastewater Program collects and displays wastewater data from communities across the United States; data is available at the site level, as well as the state, regional, and national levels, and is updated every Friday with approximately 1,500 wastewater monitoring sites reporting weekly. Wastewater monitoring is implemented in all 50 states, 7 territories, and some tribal communities. NWSS aggregates data from three streams: ELC-funded state/local health departments, Verily's national testing contract, and the academic WastewaterSCAN network. Data are freely downloadable as CSV from data.cdc.gov and available via GitHub. NWSS rapidly expanded from 209 sampling sites in September 2020 to more than 1,500 sites by December 2022, covering approximately 47% of the U.S. population. [CDC + 2](https://www.cdc.gov/nwss/index.html)

7. CDC COVID Data Tracker

This is a public-facing aggregation portal drawing from NHSN, NREVSS, NWSS, NSSP, and the National Vital Statistics System. It covers all 50 states and territories with county-level hospitalization data available for COVID-19. Influenza and RSV appear via cross-links to FluView and the Respiratory Illness Data Channel. All data are publicly downloadable via data.cdc.gov. Coverage varies by metric — some components include territories while others do not.

8. WastewaterSCAN

An academic-commercial wastewater program run by Stanford, Emory, and Verily. WastewaterSCAN monitors SARS-CoV-2, influenza A/B, RSV, and an extended panel of pathogens across Midwest, Northeast, Southern, and Western states. Sites number approximately 148+ following a ~24% contraction in mid-2024. Data are publicly available on data.wastewaterscan.org and via monthly newsletters at wwscan.ghost.io. WastewaterSCAN data are collected as part of a partnership between Stanford University, Emory University, and Verily and reported to CDC NWSS, meaning these data appear both on the WastewaterSCAN dashboard and within CDC's NWSS portal. [WWSCAN](https://wwscan.ghost.io/)

[Restoredcdc](https://restoredcdc.org/www.cdc.gov/nwss/about.html)

9. Biobot Analytics

Biobot tests wastewater nationwide for influenza A and B, RSV, and SARS-CoV-2, publishing weekly Respiratory Risk Reports with national and regional concentration trends. Biobot operates approximately 150–200 sites during respiratory season. Biobot Analytics ended its popular public dashboard due to a lack of internal resources but continues to provide weekly updates; its reports include trends from about 150 to 200 testing sites. Free weekly reports provide national and regional trends; raw site-level and state-level data require a commercial contract. Unlike WastewaterSCAN, Biobot data are not incorporated into the CDC NWSS dashboard. [Biobot Analytics](https://biobot.io/risk-reports/covid-19-influenza-and-rsv-wastewater-monitoring-in-the-u-s-week-of-july-1-2024/)

[The Sick Times](https://thesicktimes.org/2024/07/23/wastewater-surveillance-for-covid-19-keeps-evolving-heres-what-you-need-to-know/)

Key Cross-Cutting Observations

Geographic completeness: ILINet and NHSN HRD offer the broadest territorial reach (all 50 states + multiple territories). NSSP covers all 50 states + DC + Guam but suppresses some jurisdictions. RESP-NET is intentionally limited to 14 sentinel states. NREVSS reports only at national/regional levels publicly. NWSS covers all 50 states and 7 territories across ~1,500 sites.

Access tiers: All CDC systems offer at least some free public data. NSSP is the most restricted, requiring ESSENCE credentials for granular access. Biobot's commercial tier and NHSN's facility-level data both require agreements or contracts. WastewaterSCAN and NWSS are the most openly accessible at the most granular level.

External integration: The CDC Respiratory Illness Data Channel serves as the integrating layer, pulling together NHSN (hospitalizations), NSSP (ED visits), NREVSS (lab positivity), and NWSS (wastewater) into a single public view — the closest thing to a unified U.S. respiratory surveillance dashboard available to the public.

Surveillance gaps: The CDC's wastewater dashboard, while the most comprehensive source of wastewater data, may present COVID-19 levels as "low" when the virus is still spreading widely; wastewater data are an imperfect proxy for cases and cannot be directly translated into numbers of sick people. Similarly, ILINet's syndromic approach cannot distinguish between pathogens without paired laboratory data. [The Sick Times](https://thesicktimes.org/2024/07/23/wastewater-surveillance-for-covid-19-keeps-evolving-heres-what-you-need-to-know/)

**Query 2:**

I want another dashboard that looks at state health department data only.

**Output 2**:

Catalogued state health departments and architected interactive filterable dashboard

Catalogued state health departments and architected interactive filterable dashboard

Good idea. Let me build this as a fully interactive, filterable reference dashboard in the chat so you can sort and filter on the fly — no file needed.

Architected comprehensive state health surveillance dashboard with filterable data

[Table]

Here's what the dashboard covers and how to use it:

Filtering options. You can search by state name or abbreviation, filter by HHS region (all 10), filter by tier, and filter by whether a specific pathogen is tracked in state-level output. Hovering any row surfaces a notes tooltip with sourcing details.

The three tiers reflect real variation in state capacity:

- Tier 1 (8 states): California, New York, Texas, Washington, North Carolina, Virginia, Minnesota, and Wisconsin all operate dedicated, interactive multi-pathogen dashboards that cover all three viruses with multiple surveillance modalities (syndromic ED, lab, hospital, wastewater, or mortality), updated weekly, often with sub-state geographic breakdowns and downloadable data.
- Tier 2 (34 states + DC): Most states publish at least a weekly influenza surveillance report — many now incorporate COVID-19 activity levels, and some include RSV. These are often PDF/report-based rather than interactive dashboards. RSV is the most commonly absent pathogen at the state level; Florida stands out for having significantly pulled back COVID-19 and RSV public reporting since 2023.
- Tier 3 (8 states): Montana, Wyoming, North Dakota, South Dakota, Idaho, Nevada, Alaska, and Hawaii produce primarily flu-only weekly summaries and otherwise rely on CDC national systems (NWSS, NREVSS, NSSP, NHSN) for COVID-19 and RSV visibility. Geographic isolation, small populations, and limited public health informatics capacity are the common threads.

A key pattern worth flagging: RSV is the weakest link in state-level surveillance. Only the Tier 1 states consistently track it as a standalone public-facing metric. Most Tier 2 states get RSV data through NREVSS at the HHS-region level, not through their own state systems. This is partly a historical artifact — RSV was not a reportable condition in most states until recently — and partly a capacity issue.
